# Feasibility and efficacy of a multi-factorial intervention to prevent falls in older adults with cognitive impairment living in residential care (ProF-Cog). A feasibility and pilot cluster randomised controlled trial

**DOI:** 10.1186/s12877-017-0504-6

**Published:** 2017-05-30

**Authors:** Julie Whitney, Stephen H.D. Jackson, Finbarr C. Martin

**Affiliations:** 1 King’s Health Partners, King’s College Hospital, Denmark Hill, Brixton, London SE5 9RS UK; 2 King’s Health Partners, St Thomas’ Hospital, Lambeth, London SE17EH UK

**Keywords:** Fall prevention, Dementia, Exercise, Residential care, Feasibility

## Abstract

**Background:**

Falls are common in people with dementia living in residential care. The ProF-Cog intervention was developed to address fall risk factors specific to this population. The aim of this study was to evaluate the safety, acceptability, and feasibility of the intervention and provide an estimate of its efficacy.

**Methods:**

This was a cluster randomised controlled pilot study undertaken in care homes in London, UK. All permanent residents living in participating homes who were not terminally ill were invited to participate. The intervention included an assessment of falls risk factors followed by a tailored intervention which could include dementia care mapping, comprehensive geriatric assessment, occupational therapy input and twice-weekly exercise for 6 months as required to target identified risk factors. The control group received usual care without a falls risk assessment.

Standing balance was the primary outcome. This and other outcome measures were collected at baseline and after 6 months. Falls were recorded for this period using incident reports. Changes were analysed using multi-level modelling. Adherence to the interventions, adverse events and trial feasibility were recorded.

**Results:**

Nine care homes enrolled in the study with a total 191 participants (51% of those eligible); five homes allocated to the intervention with 103 participants, and four homes to the usual care control group with 88 participants. The intervention was safe with only one reported fall whilst undertaking exercise. Adherence to agreed recommendations on activity and the environment was modest (21 and 45% respectively) and to exercise was poor (41%). Balance scores (score range 0–49) analysed on 100 participants decreased by a mean of 3.9 in the control and 5.1 in the intervention groups, a non-significant difference (*p* = 0.9). In other measures, both groups declined equally and there was no difference in falls rates (IRR = 1.59 95%, CI 0.67–3.76).

**Conclusion:**

The intervention was safe but not clinically effective. Poor adherence suggests it was not an acceptable or feasible intervention.

**Trial registration:**

ISRCTN00695885. Registered 26th March 2013.

## Background

Falls are one of the most common causes of harm in older people living in residential care settings. Up to half of all residents in such settings are likely to fall each year and in the UK, 20% of all hip fracture admissions come from care homes [1, 2]. Cognitive impairment is common affecting 50–80% of those living in care homes [3] and the risk of falls is higher in the presence of cognitive impairment [4]. This is thought to be due to a combination of a higher prevalence of known fall risk factors such as gait and balance impairment and risk factors specific to cognitive impairment such as impaired attention and concentration, wandering, impulsive and agitated behaviours [5].

Interventions to prevent falls in residential care have had equivocal results with some interventions effectively reducing falls and others not [6]. One reason for this may be that some interventions tested were not adequately tailored to address the specific risk factors identified in the care home population. Our group previously investigated the risk factors associated with falls in older people with cognitive impairment living in residential care (RC) and found important risk factors for falling were slightly different to those found in community dwelling populations. Independent risk factors for falling included poor balance and gait, use of a walking frame, severity of cognitive impairment, presence of impulsive or wandering behaviours, use of psychotropic or anti-depressant medication and previous falls [7]. In a more in-depth analysis of risk; anti-depressant use, impaired balance (measured using postural sway), anxiety and impaired attention and concentration were independent and significant predictors of falling [5]. This suggests that the profile and relative importance of the many falls risk factors differs between the older community dwelling population and the population of older people resident in care homes. Specifically, postural instability responsive to exercise may be relatively more important in community populations, so that exercise interventions alone are almost as powerful as multifactorial approaches [8]. In contrast, the care home populations may have multiple risk factors including cognition and behaviour and interventions may generally need to be more broad based to address these additional risk factors. Reflecting on the risk factors identified above, this would need a multi-factorial intervention to include exercise to address gait and balance instability, medication modification review to address culprit medications, enriching the environment and optimising safe participation in functional activity to minimise the impact of impaired attention and orientation and to engender a person-centred approach to manage anxiety. This formed the basis of the ProF-Cog intervention.

A major challenge in researching interventions to prevent falls in the care home context is that the extent of cognitive impairment and physical frailty could render interventions targeting potentially modifiable risk factors, more difficult to deliver and the effects more difficult to measure. For example, there is evidence to suggest that intensive exercise interventions delivered to those with mild to moderate dementia living in the community results in fewer falls [9] but the prevalence of severe cognitive impairment and physical frailty is higher in residential care homes [10] and it is unclear whether such interventions would be feasible in these populations.

The *Prevention of falls in older adults with cognitive impairment* (ProF-Cog) study was a feasibility and pilot cluster randomised controlled trial testing a multi-factorial intervention developed to address the fall risk factors found in observational studies of this population [5]. The objectives of this study were to determine the safety, acceptability, feasibility and to estimate the clinical efficacy of the intervention with the aim of informing the design of a larger future definitive trial.

## Method

### Trial design

The study was a cluster randomised controlled trial with the unit of cluster being each participating home. Despite being a pilot study, an RCT design was used to allow detection of a clinical effect in a potentially deteriorating population, which may be an effect of maintenance rather than improvement. A clustered design was necessary as many of the components of the intervention involved the care home staff and environment.

### Participants and setting

Recruitment began in April 2013 and ended in October 2013. In the UK, care homes are independent businesses. There are two type of care home where care is available 24 h a day (24/7). Nursing homes will have a registered nurse on site 24/7 whereas residential homes are run by care staff and receive ad hoc nursing input from district nursing. Both nursing and residential care homes in South East London, UK were approached and given information about the study. Managers were asked if the home was currently enrolled in any other projects. If so, managers were asked to describe. This was followed up by the research team and if they were participating in registered research, the home was ineligible.

If the home manager agreed that the home could take part in the study, individual participants were approached for consent. If a potential participant was deemed not to have capacity to consent following a capacity assessment, personal consultees were contacted. A personal consultee is defined as an individual involved in the unpaid care of the potential participant or with an interest in the potential participant’s welfare. In the majority of cases this was the next of kin. If no personal consultee was available, nominated consultees were sought. Nominated consultees are defined as someone who is prepared to be consulted about the project but has no connection with the project [11].

Permanent residents were included in the study providing home managers thought they were likely to survive for the 6-month study period and understood the English language sufficiently to participate. Although the intervention was designed for those with cognitive impairment, it was not an inclusion criterion due to the small numbers expected to be fully intact and to optimise generalisability to the setting.

### Randomisation and blinding

Participants recruited to the trial underwent baseline assessment prior to allocation being revealed. No further recruitment took place in a home once allocation was known. All follow up measures were performed by an assessor not involved in the delivery of the intervention. It was not possible for them to be fully blinded to group allocation as they visited care homes where staff were aware of allocation. Randomisation was computer generated and stratified by the presence of nursing beds in the home. The Clinical Trials Unit at King’s College London ran the randomisation process and revealed allocation when requested by the chief investigator. Allocation was revealed in “blocks” of two homes at a time.

### Intervention

The intervention consisted of two linked processes. Firstly, all participants underwent a multifactorial fall risk assessment (MFRA) designed to identify the risk factors demonstrated in our previous prospective cohort study[5, 7]. Secondly, the “therapeutic” intervention which had two approaches: one was aimed at modifying the identified impairments of an individual participant; the other was aimed at impacting the individual’s risk by altering their environment or the care provided for them by the care home staff. Care homes randomised to usual care and their individual participants received neither the assessment nor the therapeutic interventions. Table 1 provides details of the assessments included in the MFRA to identify the risk factor and the intervention delivered to target each of the identified risk factors.

**Table 1:** Description of the ProF-Cog intervention specifying assessment and intervention to address specified fall risk factors

Risk factor	Gait and balance impairment	Medication	Medical conditions	Cognitive impairment	Behavioural and psychiatric
Risk factor identified in Whitney et al. [5]	Unable to stand 10 s with eyes closed	Antidepressant or hypnotic/anxiolytic medication	N/a	Cognitive function Attention and concentration	FIBS, GAS, NPI
How was it assessed in the MFRA?	Able to rise from a chair without assistance^a^	List of medication and doses	List of medical conditions Falls history Lying /standing BP Neurological and musculoskeletal examination	ACE-R at baseline	FIBS and NPI-NH at baseline
Recommended intervention	Gait and balance exercises Check and maintenance of walking aids Provision of 3 pairs of hip protectors	Geriatrician review (with possible CGA) if using psychotropic drugs.	Geriatrician review (with possible CGA) if Undiagnosed cognitive impairment, dizziness, unexpected falls, orthostatic hypotension, agitated or anxious behaviours.	Environmental assessment Advice of environmental safety and optimising participation on ADLs Pool activity level and advice on participation in meaningful activities	Dementia care mapping and resulting action plan. Bed and chair sensors
Intervention not required if	Bedbound Unable to rise from a chair Unable to follow very simple commands (i.e. get up from the chair)	No culprit medications.	No relevant medical conditions identified at assessment	All participants included	No evidence of impulsivity, wandering, anxiety or agitation.
Proportion offered intervention	Exercise = 69% Hip protectors = 61%	33%	49%	100%	DCM = 37% Sensors =4%
Uptake of the intervention^b^	Completed exercise = 30% Hip protectors (agreed to wear) = 46%	-	-	-	-

*FIBS* Falls related impulsive behaviour scale, *GAS* Goldberg Anxiety Scale, *NPI (−NH)* Neuropsychiatric inventory (nursing home version), *MFRA* Mutli-factorial falls risk assessment, *ACE-R* Addenbrookes Cognitive Examination Revised, *CGA* Comprehensive Geriatric Assessment, *ADLS* Activities of daily living, *DCM* Dementia Care Mapping

### Balance training

All participants who could rise from a chair without help, were not bed bound and were able to follow very simple instructions (could complete the baseline physical assessment), were offered twice weekly balance training exercise delivered by a physiotherapist or therapy assistant. It was anticipated that all residents who were able to get up without help would have some degree of balance or gait dysfunction which would benefit from exercise. Those who couldn’t stand would be at lower risk of gait or balance related falls and participation in balance training would not be feasible for that group.

Where possible, exercise took place in small groups (maximum 6) with two therapists supervising. For more physically or cognitively impaired participants who were not able to participate in group exercise, it was provided on a one to one basis. Exercise sessions were planned to last 45 min twice a week and exercise mostly performed standing up. These included moving centre of gravity (leaning, reaching), reducing base of support (tandem standing and walking) and minimising upper limb support. Exercises were based on the Otago exercise programme [12].

Walking aids were checked for safety and replaced if necessary. Suitable walking aids were provided to those who required them.

Mobile participants were provided with three pairs of hip protectors to reduce the risk of injury that could be associated with increased activity levels resulting from exercising. The hip protectors were provided as a safety precaution to mediate the potential increased exposure to falls if participants were to become more physically active. The study was not powered for and did not aim to determine their effect in preventing hip fracture.

### Geriatrician and medication review

All MFRAs were reviewed by a geriatrician to determine whether a consultation was required. Those with undiagnosed cognitive impairment, dizziness, unexplained falls, orthostatic hypotension or agitated or anxious behaviours as well as those who required medication review were visited in the care home by a geriatrician. The geriatrician reviewed each participants’ medication list and MFRA to determine whether medication review was required. Medication review would generally be prompted by use of anxiolytic, hypnotic, antidepressant or antipsychotic prescriptions and findings of dizziness or orthostatic hypotension.

### Environmental and activity assessment

The bedroom and other environments used by participants were assessed by an occupational therapist (OT). This took into account both the physical environment and each person’s limitations. Layout of the room to maximise safety, removal of trip hazards, equipment required and methods to optimise participation in meaningful functional activity were considered. All participants were screened using the Pool Activity Level (PAL) [13] to determine ability to participate in meaningful activity. The assessments were discussed with the care home staff with a view to alterations needed in the participants’ immediate environment (bedroom/bathroom etc.) or aspects of the daily care plan and activity schedule. Responsibility for carrying these recommendations rested with the care home staff.

### Dementia care mapping

Dementia-care mapping is a way of delivering person-centred care. It involves systematic observation of people with dementia to determine their well-being and the factors that enhance or detract from it. The findings are shared with staff and care plans developed based on the findings. There is some evidence that it may be a useful fall prevention intervention [14].

Participants who were identified as anxious, agitated, who wandered or were impulsive on the MFRA were observed using Dementia Care Mapping undertaken by two trained research team members. Observations of varying length were conducted to provide more understanding of these behaviours. Findings were shared with the care team who then worked with the intervention team to develop individualised recommendations.

### Bed and chair sensors

Participants who scored highly on the fall related impulsive behaviour scale (FIBS)[15] at baseline and additionally had very poor standing balance (unable to stand for 10 s without support) were provided with bed and chair sensors. These are intended to provide an alert to care home staff so that they might take action to avert the risk of the participant falling when moving without supervision.

### Professional training and awareness

Training sessions were not a mandatory part of this intervention but were offered to each home at the beginning of the 6-month intervention period. Staff in each home were asked to identify gaps in their knowledge and training was provided. Examples of training sessions included person centred care, meaningful activities, seating for comfort, medications and continence. Other specialists were invited to deliver sessions where the team did not have the required expertise.

Debrief sessions were also offered on a monthly basis where staff could discuss the circumstances underlying falls in the home and what might be done to prevent further such falls.

### Usual care group

The homes randomised to receive usual care, continued as usual with no change to routine practice.

### Outcomes

All measures were performed by a research physiotherapist in the care home setting and taken once at baseline and again at follow up after 6 months.

The primary outcome measure for this study was the standing balance rating scale. This was used because balance function is a good predictor of fall risk [1] but requires a smaller sample size to detect a clinically significant difference (see Campbell et al. for clinically and statistically significant differences in balance following an exercise intervention [16]).

### Standing balance rating scale

The participant was asked to stand first with their feet apart and without upper limb support for 10 s and then progressed to standing with feet together, semi-tandem stand, tandem stand and single leg stand, holding each position for 10 s. If a posture could not be sustained unsupported for 10 s, the test was halted and not progressed [17].

### Falls

A fall was defined as “an unexpected event in which the participants come to rest on the ground, floor, or lower level” [18]. Falls data were regularly collected from each care home for the 6 months from baseline using care home reporting mechanisms.

### Other measures

Other measures were included in this study to determine their feasibility to use in a larger trial where they would be used to; analyse confounding effects, assess cost efficacy and to describe the cohort of the trial.

The timed up and go [19], grip strength and sit to stand ability, the EQ5D [20], iconographical FES-I [21], Cornell depression scale [22] and Addenbrooke’s Cognitive Examination (ACE-R) [23] were taken at baseline and follow up. Care staff with knowledge of the resident were questioned using the NPI-NH [24], Barthel [25], Physical activity in residential care (PAM-RC) and the Cornell depression scale. Impulsivity was measured at baseline using the falls related impulsivity scale (FIBS) [15].

#### Safety

Falls and adverse events that could be attributed to the intervention were recorded.

#### Uptake and adherence

Uptake and attendance at exercise sessions and reasons for non-attendance were collected for the duration of the intervention. Individual participant engagement was rated in exercise sessions once weekly on a scale of 0–10 with 0 being completely disengaged with the intervention to 10 being fully engaged. This rating is a based on the therapists’ judgement of how they perceived the participant to engage and is an intuitive rating that gives a sense of the effort and enthusiasm with which a participant takes part in the exercise. This scale has been previously tested for inter-rater reliability which was good = ICC 0.82 (95%CI0.76–0.87) [26].

Where recommendations were made about changes to the environment, activity provision or based on dementia care mapping; staff adherence was recorded by the occupational therapist based on their impression, using a rating of “followed fully”, “followed partially” or “not followed at all” and the same engagement rating scale was used as for the exercise sessions to rate the engagement of the staff with these processes.

#### Trial feasibility

Feasibility of outcome measures used was assessed by recording reasons for non-completion as well as a rating of average time taken to perform. Finally, the testers rated their difficulty completing the test in this population (rated 0–10, zero being not at all difficult).

### Sample size calculation

Since this was a pilot study, clinical effects were only one component of a trial which would ultimately use falls as a primary outcome. One of the purposes of this study was to determine the sample size needed for a definitive trial. Therefore, sample size calculations involved some estimation.

A sample size of 46 participants per group was thought to be sufficient to detect an improvement of 0.8 s in the standing balance rating scale (baseline 1.7, SD = 13 – data from previous unpublished pilot work) in the intervention group. A design effect of 2 (estimated) was used to adjust for clustering increasing this to 92. Taking into account a 15% drop out rate (estimated) 106 participants per group (212 in total) in 6 clusters (n ~ 35) were required. Design effect and attrition rates were based on those used in a similar study [27].

### Statistical methods

Baseline data were compared for between group differences using parametric or non-parametric analyses as appropriate. Since this was a feasibility study, reasons for missing data were analysed using descriptive statistics but imputation of these data was not performed due to large proportions of missing (not at random) data in some variables.

The primary outcome measure of standing balance was analysed by calculating the change in scores between baseline and follow up and using multilevel modelling adjusting for baseline score as well as care home to account for cluster effects. A comparison of models created with and without using the intervention as a second independent variable was made using the -2LL statistic. For the analysis of balance scores, those who had a standing balance score of 0 both at baseline and follow up (i.e. could not stand at all on either occasion) were removed from the dataset.

Other variables were analysed in the same way to look for between group differences from baseline to follow up but scores of 0 were not excluded from analysis.

Falls data were analysed using negative binomial regression analysis adjusting for clusters.

To provide data for further definitive trials, the intracluster correlation coefficients (p) and design effects (DE) were calculated from each baseline variable using the following formula $p=\frac{\mathrm{Sb}2}{Sb2+ Sw2}$ where S^b2^ is variance between clusters and S^w2^ the variance within clusters. Design effect t was calculated *DE* = 1 + *p* (*m* − 1) where m = mean cluster size.

Data were analysed using SPSS version 20 and STATA version 12.

## Results

### Recruitment

**Fig. 1:**
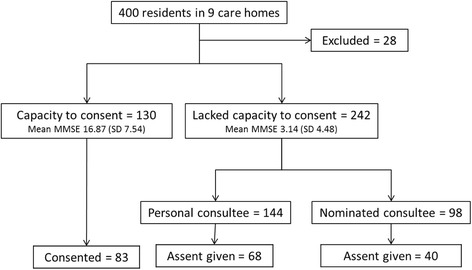
Detail of recruitment to the study

Fifteen care homes were identified and approached. Three homes declined participation, one due to having an interim manager, one was in the process of closing down and another did not give a reason. Three were ineligible due to existing research commitments. The nine care homes who agreed to participate looked after 400 potential participants (filled beds at the commencement of recruitment). Of these 130 (33%) were judged to have capacity to decide whether to participate in the study. A total of 191 were recruited to take part in the trial. Reasons for exclusion included death before recruitment (*n* = 5), temporary residence (*n* = 4), insufficient English language (*n* = 4) and life expectancy of <6 months (*n* = 15) (Fig. 1). Over half of those approached to participate declined to take part. The enrolment rates differed dependent on capacity to consent to take part with 35% of those with capacity declining compared to personal consultees declining in 53% and nominated consultees in 59% of cases (Fig. 1).

### Baseline data

**Fig. 2:**
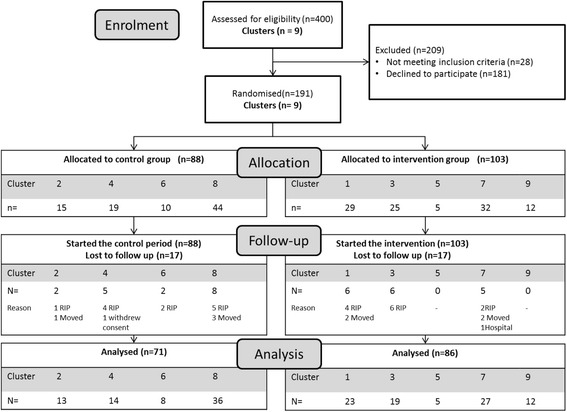
Consort diagram for study

Four nursing homes and five residential homes took part in the study. See Fig. 2 for study consort diagram. The 191 participants had a mean age of 83.5 (SD8.8) and 69% were female. Most participants were cognitively impaired, the mean ACE-R was 28.3 (SD26.2, range 0–91) and only 6 (3%) had a score of ≥80 suggesting they were cognitively intact. When participants from intervention and control groups were compared, the intervention group were found to have significantly more medical conditions and took longer to complete the timed up and go test. They were also less likely to be in a care home with 24/7 nursing (a nursing home) and there was a trend towards more of the intervention group having fallen in the previous year (Table 2).

**Table 2:** Comparison of baseline variables

Measure (number for which data was collected)	Control (*N* = 88)					Intervention (*N* = 103)						P
	Total	Cluster				Total	Cluster					
		2	4	6	8		1	3	5	7	9	
	N (%)	15	25	10	44	N (%)	29	25	5	32	12	
Home with nursing	63 (72%)	0	19 (100%)	0	44 (100%)	57 (55%)	0	19 (100%)	0	32 (100%)	0	0.02
Female	60 (68%)	11 (73%)	13 (68%)	10 (100%)	26 (59%)	72 (70%)	22 (76%)	16 (64%)	3 (60%)	19 (60%)	12 (100%)	NS
Fall in the past year	36 (41%)	8 (53%)	5 (26%)	3 (30%)	20 (46%)	56 (54%)	18 (62%)	11 (44%)	3 (60%)	15 (47%)	9 (75%)	0.06
	Mean (SD)					Mean (SD)						
Age in years	83.0 (9.2)	85.2 (8.6)	82.0 (8.7)	89.7 (6.1)	80.2 (9.6)	84.1 (8.4)	84.6 (8.1)	84.4 (9.6)	88.4 (3.4)	81.1 (9.6)	86.7 (5.3)	NS
Years in the home	2.7 (3.1)	2.3 (1.9)	2.3 (2.1)	7.1 (5.7)	2.3 (3.0)	2.3 (2.4)	2.2 (2.2)	2.7 (2.5)	2.2 (3.1)	2.1 (2.9)	2.4 (2.2)	NS
Number of medications	8.0 (3.7)	7.4 (3.1)	10.1 (4.3)	8.7 (2.7)	7.1 (3.5)	8.0 (4.1)	7.6 (4.4)	7.7 (3.6)	5.6 (4.7)	9.3 (4.4)	7.8 (3.4)	NS
Number of medical conditions*	1.5 (1.2)	1.1 (1.2)	2.1 (1.1)	1.5 (1.0)	1.5 (1.2)	2.0 (1.4)	1.6 (1.3)	2.2 (1.3)	1.6 (2.1)	2.5 (1.5)	1.1 (0.8)	0.03
ACE-R (*n* = 171)	27.2 (27)	34.1 (23.6)	33.0 (27.1)	19.8 (33.5)	24.1 (25.9)	29.1 (26)	31.6 (24.5)	22.9 (23.5)	32.0 (34.8)	38.0 (27.2)	31.9 (30.4)	NS
Barthel (*n* = 189)	9.4 (5.8)	14.4 (2.9)	7.9 (5.7)	6.1 (4.3)	9.4 (5.9)	8.6 (5.9)	11.2 (5.0)	6.3 (5.7)	12.4 (3.4)	5.8 (5.1)	12.8 (5.7)	NS
Health today (*n* = 90)	70.2 (30)	76.3 (32.2)	53.6 (25.4)	81.7 (11.5)	71.6 (32.4)	65.4 (24)	65.5 (22.2)	63.3 (30.5)	95.0 (10)	62.1 (11.5)	57.9 (24.1)	NS
FES-I (*n* = 80)	17.4 (7.2)	18.1 (7.6)	25.0 (4.6)	23.5 (0.7)	12.7 (4.4)	18.5 (8.2)	18.2 (6.9)	25.8 (5.7)	8.0 (2.0)	20.2 (8.1)	13.8 (8.3)	NS
PAM-RC (*n* = 188)	10.4 (6.4)	12.4 (3.7)	7.9 (6.7)	9.3 (5.1)	11.1 (7.0)	9.2 (6.3)	12.0 (5.8)	8.0 (6.1)	13.6 (4.8)	5.5 (4.8)	14.0 (5.1)	NS
Cornell resident (*n* = 111)	3.8 (5.0)	4.9 (6.6)	4.9 (6.8)	2.0 (2.0)	2.9 (2.6)	4.9 (4.8)	5.9 (4.7)	5.1 (5.5)	0 (0)	5.1 (4.1)	4.3 (6.2)	NS^b^
Cornell carer (*n* = 188)	3.7 (3.9)	2.7 (2.6)	4.4 (4.5)	1.5 (1.8)	4.1 (4.3)	3.4 (3.7)	5.6 (5.0)	3.0 (3.4)	2.6 (2.7)	2.3 (1.9)	2.0 (2.0)	NS^b^
FIBS (*n* = 189)	2.2 (3.1)	1.4 (1.8)	2.5 (3.9)	1.6 (2.2)	2.4 (3.3)	2.0 (3.1)	2.8 (3.4)	2.1 (3.6)	2.6 (2.1)	1.3 (2.8)	1.4 (2.3)	NS^b^
NPI-NH (*n* = 191)	11.5 (12)	6.3 (7.0)	15.6 (16.1)	5.7 (7.2)	12.8 (12.1)	10.1 (10)	13.8 (13.0)	7.4 (8.9)	7.0 (8.0)	10.1 (9.7)	7.9 (7.1)	NS^b^
Balance score (*n* = 150)	13.8 (12)	19.4 (11.1)	10.1 (10.8)	7.8 (10.2)	15.1 (12.3)	13.7 (14)	16.9 (12.8)	9.2 (12.3)	24.2 (13.1)	8.4 (14.1)	22.9 (14.0)	NS
Timed up and go (*n* = 92)	37.6 (29)	34.3 (24.0)	34.1 (11.3)	51.8 (30.0)	37.7 (36.3)	61.8 (55)	55.7 (47.5)	60.3 (38.6)	31.0 (10.1)	105.5 (82.3)	38.6 (29.0)	0.005^a^

^a^Difference analysed using log transformed data

^b^Non-parametric data analysis carried out as log transformation did not alter skew / data was not interval or ratio level

^*^Total from the following medical conditions: Depression, Parkinson’s disease, Stroke, Hypertension, Myocardial infarction, Heart failure, COPD, Hip fracture, Osteoarthritis, Epilepsy, Diabetes

### Outcome measures

There was no significant difference between balance score changes in the intervention compared to the control group. With the exception of a significant increase in staff rated Cornell depression scores in the intervention group, no other secondary outcome measure differed between the two groups. In fact, the whole cohort demonstrated a significant decline in function (modified Barthel Index), strength (sit to stand), mobility (timed up and go), balance score, cognition and mood over the 6-month follow up (Table 3). Excluding the six participants (4 intervention, 2 usual care) with an ACE-*R* ≥ 80 (cognitively intact) did not change these outcomes (Table 4). Neither did repeating the analysis on males and females separately, nor in those either with or without the highest level of physical performance. High physical performance was defined as performance in the highest quartile on two or more of the following; timed up and go, grip strength or balance (*N* = 26).

**Table 3:** Differences between the intervention and control group (all participants)

	Control (*N* = 71)	Intervention (*N* = 86)	-2LL				Significance p=	ICC (DE)
Change scores	Mean (SD)	Mean (SD)	Without intervention/control	With intervention/control	Change in -2LL	df change	^a^	
Primary outcome measure								
Balance score (*n* = 100)	−3.90 (9.68)	−5.14 (9.63)	470.31	470.11	0.21	2	0.90	0.75 (15.9)
Other outcome measures								
ACE-R (*n* = 136)	−1.76 (12.63)	−5.90 (9.93)	1019.5	1015.0	4.5	2	0.11	0.37 (8.4)
Health today (*n* = 62)	3.83 (35.80)	2.24 (31.74)	574.08	571.97	2.11	2	0.35	0.57 (12.4)
FES-I (*n* = 49)	−3.57 (5.73)	−1.86 (4.35)	291.44	290.42	1.02	2	0.60	0.8 (17.7)
PAM-RC (*n* = 156)	0.69 (3.74)	−0.88 (3.27)	825.1	823.7	1.4	2	0.50	0.82 (17.5)
Cornell resident (*n* = 86)	1.76 (5.43)	1.56 (4.29)	489.77	489.72	0.05	2	0.97	0.52 (11.4)
Cornell carer (*n* = 155)	−0.27 (4.08)	1.0 (4.61)	805.43	797.78	7.65	2	0.02	0.74 (15.8)
NPI-NH (*n* = 157)	−1.69 (13.96)	0.96 (11.32)	1151.66	1148.39	3.27	2	0.20	0.66 (14.2)
NPI – disruptiveness (*n* = 157)	−0.43 (4.51)	−0.81 (2.91)	738.9	736.7	2.21	2	0.33	0.55 (12.0)
Sit to stand score (*n* = 110)	−0.02 (0.81)	−0.08 (0.87)	255.2	254.5	0.69	2	0.71	0.85 (18.1)

^a^Analysed using multilevel model adjusting for clustering based on care home. The change from baseline to follow up was the dependent variable and the baseline value the independent variable

**Table 4:** Differences between the intervention and control group (those with ACE-*R* < 80)

	Control (*N* = 86)	Intervention (*N* = 99)	-2LL				Significance p=
Change scores	Mean (SD)	Mean (SD)	Without intervention/control	With intervention/control	Change in -2LL	df change	^a^
Primary outcome measure							
Balance score (*n* = 95)	−4.00 (9.77)	−5.22 (8.00)	436.12	435.57	0.55	2	0.76
Other outcome measures							
ACE-R (*n* = 131)	−1.51 (12.6)	−5.82 (10.2)	983.03	978.15	4.89	2	0.09
Health today (*n* = 57)	4.68 (36.2)	3.55 (33.3)	523.99	522.90	1.10	2	0.58
FES-I (*n* = 45)	−3.57 (5.7)	−1.92 (4.7)	268.00	267.86	0.14	2	0.93
PAM-RC (*n* = 150)	0.57 (3.8)	−0.88 (3.3)	798.39	797.00	1.39	2	0.50
Cornell resident (*n* = 81)	1.75 (5.5)	1.44 (4.1)	460.73	460.73	0	2	1.0
Cornell carer (*n* = 149)	−0.30 (4.1)	1.20 (4.5)	776.38	768.48	7.91	2	0.019
NPI-NH (*n* = 151)	−1.74 (14.1)	1.63 (10.9)	1108.99	1105.75	3.25	2	0.20
NPI – disruptiveness (*n* = 151)	−0.44 (4.6)	−0.56 (2.5)	713.57	711.67	1.90	2	0.39
Sit to stand score (*n* = 105)	−0.02 (0.8)	−0.13 (0.8)	240.26	239.31	0.96	2	0.62

^a^Analysed using multilevel model adjusting for clustering based on care home. The change from baseline to follow up was the dependent variable and the baseline value the independent variable

There were 119 falls in total in the 6 month follow up period with 25 (28%) people in the control group having 41 falls and 31 (30%) of the intervention group falling 78 times over this period. There was no significant difference in risk of being a faller (RR = 1.09 95%CI0.58–2.03) or the rate of falls (IRR = 1.59 95%CI 0.67–3.76) when adjusted for clustering. There were 18 injuries sustained from falls including 4 fractures and 1 head injury. There was no significant difference in risk of sustaining an injury between the intervention and control group (11% and 8% respectively), RR = 1.20 (95%CI 0.66–2.20).

### Safety

One person fell whilst taking part in a one to one exercise session (0.006% of total sessions). No injury was sustained. No other intervention related adverse effects were noted over the course of the trial.

### Uptake and adherence

Details of the proportions of interventions offered to individual participants and uptake of those interventions are provided in Table 1.

#### Environment and activity

An environmental assessment and activity checklist (PAL) was completed by the occupational therapist for most of the intervention group (90% and 92% respectively). Reasons for non-completion were death or hospital admission prior to assessment. Most residents had at least one observed environmental risk factor with a mean of 3 risk factors identified per resident. Risk factors related to the bed were noted in three quarters of the residents. Common bedside risk factors included inappropriate bed rails, lack of a bed lever, unsuitable mattresses, lack of a night light, bedside table or call bell not within easy reach.

One hundred and ninety-nine issues were discussed with the care staff concerning the environment. Less than half of the agreed recommendations (45%) were followed fully and more than a third (37%) were not followed at all. The mean engagement rating for this work was 8/10. Only 21% of the agreed recommendations on activity following the PAL were followed fully and the mean engagement score for this was 8/10.

#### Dementia care mapping

Thirty-eight of the intervention group fulfilled the criteria for dementia care mapping (DCM). Most participants required more than one observation session with 23 (60%) having 2 sessions. The average time spent on a mapping session was 1 h (61.6 min) ranging from 15 to 235 min sessions. Most of the (74%) agreed changes to care plans following DCM were “partially followed” with care staff engagement in DCM rated as 7/10.

#### Exercise

Of the 103 intervention participants, 71 were eligible to participate in exercise. Twenty-five participants were ineligible because they could not stand from a chair. Mean attendance at the exercise sessions was 41%. The most common reason, accounting for 890 (45%) missed sessions was that the participant did not wish to attend. The second most common reason was declining or fluctuating physical or cognitive functioning deeming participation in a session not possible (*N* = 405 (21%). At the end of the 6-month intervention, 31 participants (44%) were still regularly participating in the exercise programme.

The mean engagement score for participants over the duration of the exercise programme was 5/10.

#### Outcome measures feasibility

There was significant variability in the completeness of outcome measure collection. The measures which were feasible to collect (had a high level of completion and were rated as easy and quick) included questions answered by carers such as impulsivity, physical activity (PAM-RC) and function (Barthel). None of the physical tests or the participant questionnaires could be completed by more than 60% of the participants with exception for the sit to stand rating scale. The main reason for missing data was impaired physical and cognitive functioning (Table 5).

**Table 5:** Completion of baseline measures

	N (%)						0–10	Minutes
	Completed at baseline	Unable (cognitive)	Unable (physical)	Unable (cognitive and physical)	Refused	Missing other reason	Rating of ease	Time taken to complete
Physical								
Sit to stand score	158 (83%)	12 (6%)	N/a	9 (5%)	6 (3%)	6 (3%)	2	7
Balance score	100 (52%)	17 (9%)	50 (26%)^a^	12 (6%)	8 (4%)	4 (2%)	2	5
Timed up and Go	92 (48%)	15 (8%)	50 (26%)	18 (9%)	11 (6%)	5 (3%)	5	10
Near tandem standing	33 (17%)	25 (13%)	100 (52%)^a^	18 (9%)	10 (5%)	5 (3%)	7	7
5× sit to stand score	28 (15%)	19 (10%)	111 (58%)	19 (10%)	9 (5%)	5 (2%)	4	10
Question to participant								
ACE-R	135 (71%)	42 (16%)^a^	2 (1%)	5 (3%)^a^	10 (5%)	8 (4%)	6	30
Health today	110 (58%)	42 (22%)	3 (1.5%)	3 (1.5%)	17 (9%)	16 (8%)	4	8
Cornell resident	111 (58%)	47 (25%)	1 (0.5%)	3 (1.5%)	13 (7%)	16 (8%)	6	15
FES-I	76 (40%)	51 (27%)	16 (8%)	16 (8%)	18 (9%)	14 (8%)	6	12
Question to carer								
NPI-NH	191 (100%)	-	-	-	-	-	6	20
PAM-RC	188 (98%)	-	-	-	-	3 (2%	3	5
Cornell carer	188 (98%)	-	-	-	-	3 (2%)	5	15

^a^given a score of 0 if unable to do this test

## Discussion

ProF-Cog was a feasible trial to recruit to and the intervention was safe. However, adherence by care home staff and by individual resident participants was fairly low. It had no observable effect on balance, falls or any of the other outcomes.

### Feasibility of the trial and the intervention

Whilst this trial almost achieved its recruitment target in a 6-month period, a significant proportion of residents in each participating home did not take part. The group most likely to enrol was those with capacity to consent. This may reflect a general reluctance of personal/nominated consultees to make decisions about research participation on behalf of another person. Our uptake rates for those with capacity were similar to those found by Goodman et al.[28] while those without capacity similar to Zermansky et al. [29]. Further research is required to understand the process of decision making by consultees in order to optimise participation in such trials.

Some of the outcome measures proved too difficult to perform in a sufficient proportion of participants to be useful in contributing to analysis of effect. This study has provided information on which measurement tools are feasible in this setting. Measures that require any level of physical or cognitive performance will be difficult to collect from all participants and the more complex the test, the lower the completion rate. The most feasible measures were questionnaires filled in by care staff. In this study, staff were specifically chosen to complete assessments based on their knowledge of the participant. However, using staff questionnaires still carries the risk that the answers provided do not reflect the participant’s experience.

Recruiting and randomising based on care home was necessary for this trial. However, due to the small number of clusters and what turned out to be large intra-cluster effects; the randomisation did not result in two entirely homogeneous groups for comparison. There were sufficient measures in which the intervention and control group were different at baseline to suggest that the two groups were not homogenous. The reasons for the differences are not clear. The intervention group had more participants in “residential” care, but contrary to what would be expected, they appeared to be physically frailer, performing worse in the timed up and go measure. A larger trial with more or smaller sized clusters may result in more even matching.

### Effect of the intervention

This intervention did not have any effect on the outcome measures used. In fact, there was deterioration in all measures as we had predicted which was why a pre-and post-comparison without a control group would be misleading in this context. The trial was not powered for sub-group analysis, neither was this planned a priori. However, since this was a pilot study, designed to determine whether a future definitive trail would be worthwhile, knowing whether there were any trends in specific groups was considered valuable. However, no group responded differently to the intervention with balance scores deteriorating in males and females and in the frail and the fittest.

The carer reported Cornell scale scores (for participant depressive symptoms) significantly increased in the intervention group at follow up. This may be a chance finding but it is possible that being part of the intervention group and participating in activity assessment and dementia care mapping, raised staff awareness of participants’ wellbeing and therefore the follow up Cornell scores reflected a better understanding of mood.

There are several possible reasons why the intervention did not result in any change. These include; that 1) the intervention was not adequately tailored to address the risk factors, 2) the risk factors in question were not amenable to change, 3) the intervention could have been effective but was limited by poor uptake and adherence or 4) limitations in measurement was the reason for not detecting a meaningful change.

### Inadequately addressing risk factors

The ProF-Cog intervention was carefully designed to address specific fall risk factors, previously identified through prospective cohort studies, in those living in residential care. The outcome measures used in this study measured the effect of the intervention at the risk factor level. Since no effects were observed, it could be concluded that the intervention did not adequately modify the identified risk factors.

Pitkala and colleagues tested an exercise programme that reduced falls in people with mild-moderate cognitive impairment (mean mini mental state examination (MMSE) =18) [9] but the dose provided (100 + hours) was approximately double that needed for a cognitively intact, community dwelling population [30]. Considering that a higher dose may be needed, even in those with moderate, not severe cognitive impairment and leaving the poor adherence aside, the dose planned by ProF-Cog was probably insufficient. Hauer et al. (2012) also found that people with moderate cognitive impairment (mean MMSE = 22) demonstrated improved strength and function following a motor training programme [31]. The mean baseline MMSE of the ProF-Cog cohort (mean MMSE = 10) was significantly lower than these two trials suggesting a possible cognitive threshold for responsiveness to exercise. The two trials stated above recruited participants who were mostly still living in their own homes, suggesting they were also less physically frail. More research is needed to unpick the relative contribution of cognitive and physical frailty on the efficacy of exercise.

Becker et al. conducted a multi-factorial intervention in nursing homes which contained an exercise component which effectively reduced falls. The population in this study is comparable and the intervention similar in that it was multi-factorial and included exercise, environmental adaptation and staff training but no intervention aimed at addressing impulsivity and anxiety or medical management [32]. However, the effect of their intervention on falls was most apparent in between 9 and 12 months from baseline and a difference between intervention and control groups was not evident until 6 months. Therefore, it is possible that the duration of the ProF-Cog intervention was not long enough to result in changes in physiology but also in staff attitudes and understanding.

### Efficacy limited by uptake and adherence

Exercise, the most likely component of this intervention to impact on the primary outcome measure, (although possibly not on falls rates in this population), was hampered by poor adherence and high rates of attrition so that the actual dose was probably too low. In community dwelling older people, challenging balance exercise should be carried out for at least 50 h (e.g. 1 h twice a week for 6 months) in order to effectively reduce falls[30]. One third of the ProF-Cog intervention cohort were excluded from exercise and out of those who did start, half had dropped out by the end of the 6-month period and the mean attendance at sessions was 41%. This equates to 21 sessions. Even if these sessions had lasted for 1 h, this would not have achieved even half the dose of 50 h. Significant changes in balance outcome measurements probably require smaller doses of exercise than those required to influence falls rates. Nevertheless, the actual dose received was smaller than similar exercise interventions where changes in balance have been identified [33].

Exercise was only one component in the intervention. The data collected on engagement with care home staff for other interventions was generally good, but some of the recommendations particularly responding to dementia care mapping and activity provision were not widely adopted. There were many reasons for this. Staff turnover during the 6-month project was high, meaning that any training and discussions with staff at the beginning had to be repeated over the 6-month intervention period. The complexity of the intervention and the requirement for a significant change of philosophy with regard to rehabilitation and activity may have been a factor explaining the discrepancy between intention and action. Complex interventions in care homes require; readiness for involvement including support from managers, a tailored approach to each home and work to be planned jointly, with an emphasis on building relationships between the visiting healthcare professionals and care home staff [34]. While the ProF-Cog intervention adhered to some of these principles, a greater focus on these may have improved the engagement of staff with this programme.

### Limitations in measurement

Some of the measures used in this study were not feasible to use due to the low completion rates. Data collected from questioning carers was by far the most feasible to collect but better understanding of how to collect measures of physical function and quality of life from proxies and how accurately those answers reflect the reality of the experience for the person with cognitive impairment is required. The most feasible method for measuring the efficacy of an intervention in this setting is to use hard endpoints such as injurious falls including fractures or hospital admissions, but these are few in number and so the trials would require considerably more participants. In addition, greater numbers are needed because of likely intra-cluster correlations.

## Conclusion

The ProF-Cog intervention was carefully designed based on knowledge of risk factors for falls in people with cognitive impairment living in residential care. This intervention was safe but no clinical effect was identified. While it is likely that this intervention did not make any difference to fall risk factors, a lack of effect could be explained by missing primary outcome data collection reducing the sample size to below that required to detect an effect. In addition, limited uptake and adherence to some components of the intervention could explain why risk factors were not modified. The poor adherence, particularly of the exercise component questions the acceptability and therefore feasibility of this intervention in its current format. Providing specialist exercise interventions in this population didn’t result in any changes in physical function because most of this group either didn’t want to do it or were limited by their health status. Although there is evidence that some of those in a care home population wish to take part and can benefit from participating in exercise, more research is required to identify factors that contribute to favourable outcomes and how to optimise long-term adherence to exercise.

## Abbreviations

ACE-R: Addenbrooke’s Cognitive Examination –Revised
DCM: Dementia care mapping
EQ5D: Euroquol 5 dimensions questionnaire
FES-I: Falls efficacy scale international
FIBS: Falls related impulsive behaviours scale
IRR: Incidence rate ratio
MFRA: Multi-factorial falls risk assessment
MMSE: Mini mental state examination
NPI-NH: Neuropsychiatric inventory – nursing home edition
OT: Occupational therapist
PAL: Pool Activity Level
PAM-RC: Physical activity and mobility in residential care scale
ProF-Cog: Prevention of falls in older people wit cognitive impairment living in residential care
RCT: Randomised controlled trial
RR: Relative risk
